# 1H-MRS of the anterior cingulate cortex in obsessive-compulsive disorder: metabolic abnormalities in pgACC - controlled study

**DOI:** 10.1192/j.eurpsy.2023.295

**Published:** 2023-07-19

**Authors:** E. Nosková, I. Fajnerová, D. Pajuelo, P. Stopková, J. Horáček

**Affiliations:** 1 anxiety department; 2 NIMH: National institue of mental health, Klecany; 3 MR Unit, Department of Diagnostic and Interventional Radiology, Institute for Clinical and Experimental Medicine, Prague; 4 anxiety department; 5 Center for advanced studies of brain and consciousness, Institute for Clinical and Experimental Medicine, Klecany, Czech Republic

## Abstract

**Introduction:**

Obsessive-compulsive disorder (OCD) is connected with increased activity in cortico-striatal-thalamic-cortical (CSTC) loop. The anterior cingulate cortex (ACC) is a part of this loop and plays a role in error detection and monitoring and processing of conflicting information, core OCD clinical signs. This area also contains a high density of Von Economo neurons. Biochemistry in this area is closely connected with the pathophysiology of OCD.

**Objectives:**

Decreased concentrations of total N-acetylaspartate (tNAA) have been reported in ACC in patients with OCD compared to healthy controls (HC), with increase after successful treatment. Findings by other metabolites: choline-containing compounds (tCho), total creatine (tCr) and myo-inositol are not consistent. Differences in levels of tNAA, tCho, tCr would correlate with the severity of symptoms measured by Y-BOCS. In the comparison in the subgroup of patients with/ without medication, there will be differences in levels of metabolites.

**Methods:**

54 patients diagnosed with OCD according to ICD-10 and DSM-IV criteria, and 54 HC matched for age and sex were included in the study. They underwent MRI and MR Spectroscopy on a 3T Magnetom Prisma scanner (Siemens, Germany) equipped with a 64-channel volume head coil (Fig. 2). After spectral quality control, 28 OCD and 28 HC subjects were included in the statistical analysis. OCD subjects were interviewed using the Y-BOCS to evaluate the severity of the symptoms. Patients enrolled in the study were without medication at least 5 days before MRI or on a stable dosage of SSRI antidepressants. To assess the intergroup differences Wilcoxon Rank Sum test or Kruskal-Wallis test was used as appropriate. The correlation between metabolite levels and clinical characteristics was assessed by Spearman’s rank correlation coefficient. The statistics were calculated using R, version 4.1.1.

**Results:**

We found no differences in levels of tNAA in ACC in OCD vs. HC (p=0,21; see Tab.1, Fig.1). We found significantly increased level of tCho, tCr and Ins in OCD vs. HC (p=0,03; p=0,004 resp.; p=0,017 resp.). tCr levels corelated negatively with YBOCS compulsions subscale (p=0,046; cor=-0,38). tCho levels showed a trend to negative correlation with Y-BOCS compulsions subscale (p=0,067; cor=-0,35). Analysis on the subgroup with (13 subjects, 46,43 %) and without (13 subjects, 46,43 %) stable SSRI medication did not reveal significant differences.

**Image:**

**Figure:**
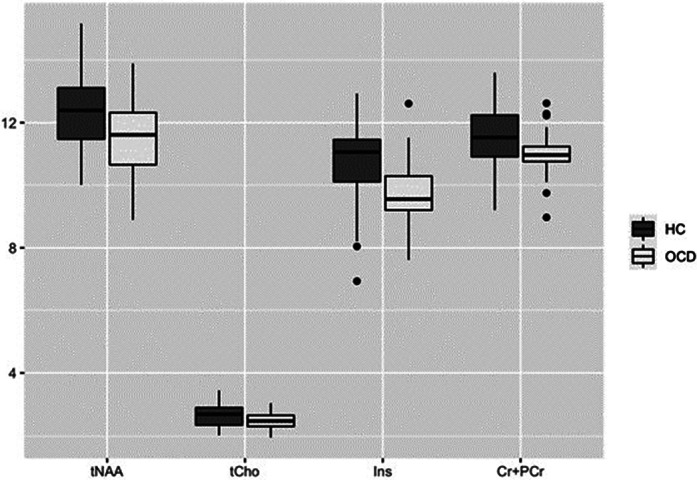

**Image 2:**

**Figure:**
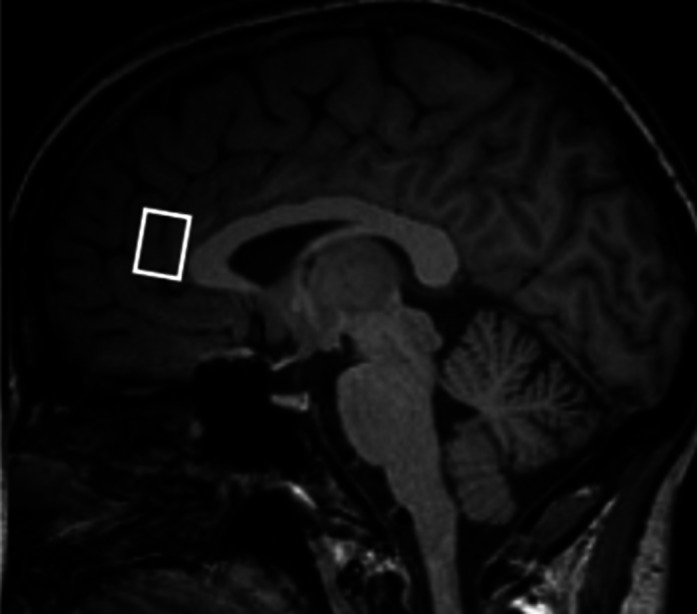

**Conclusions:**

Our study found difference in ACC by OCD patients compared to HC, mainly increased tCho, tCr and Ins. Also, the study shows a significant correlation between the severity of compulsions and tCr levels. We can see this trend also in the correlation of the tCho and Y-BOCS compulsions subscale. Similar tNAA level by OCD and HC groups could indicates correctly adjusted medication or stable state by enrolled patients.

**Disclosure of Interest:**

None Declared

